# Optimizing the Composition of Geopolymer Composites Incorporating Secondary Aluminium Industry By-Products Using Mathematical Modelling

**DOI:** 10.3390/ma18122840

**Published:** 2025-06-16

**Authors:** Artem Sharko, Van Su Le, Oleksandr Sharko, Dmitry Stepanchikov, Pavel Srb, Michal Petrů, Petr Louda, Petro Movchan, Katarzyna Ewa Łoś

**Affiliations:** 1 Institute of New Technologies and Applied Informatics, Faculty of Mechatronics, Informatics and Interdisciplinary Studies, Technical University of Liberec, Studentská 1402/2, 46117 Liberec, Czech Republic; 2 Department of Machine Parts and Mechanism, Faculty of Mechanical Engineering, Technical University of Liberec, Studentská 1402/2, 46117 Liberec, Czech Republic; longsuvp90@gmail.com (V.S.L.); pavel.srb@tul.cz (P.S.); michal.petru@tul.cz (M.P.); 3 Department of Transport Technologies and Ship Repair, Kherson State Maritime Academy, 73000 Kherson, Ukraine; avssharko@gmail.com (O.S.); mpv01121988@gmail.com (P.M.); 4 Department of Energetics, Electrical Engineering, and Physics, Kherson National Technical University, 73008 Kherson, Ukraine; dmitro_step75@ukr.net; 5 Polytechnic Faculty, University of Kalisz, Nowy Świat Str. 4, 62-800 Kalisz, Poland; petrlbc1@seznam.cz

**Keywords:** optimization, composition, geopolymers, structure, technology, properties, renewable raw materials

## Abstract

Geopolymer composite materials are a viable alternative to conventional construction materials. The research problem of geopolymer composites revolves around the imperative to comprehensively address their synthesis, structural performance, and environmental impact. The derived mathematical model facilitates precisely determining the optimal proportions of two crucial constituents in the geopolymer matrix: silica sand and secondary aluminum by-product. A mathematical model for optimizing the composition of geopolymer composites has been developed based on the integrated use of Markov chains, criterion methods, and an orthogonally compositional plan. The optimal composition of the geopolymer matrix is determined and predicted using a mathematical model. Specifically, the recommended content mixing ratio is as follows: metakaolin at 1000 g, activator at 900 g, silica fume at 1052.826 g, carbon fibre at 10 g, and secondary aluminum by-product at 62.493 g. This study analyzes the influence of different secondary aluminum industry by-products on the geopolymerization process and assesses the mechanical, thermal, and environmental properties of the resulting composites to establish a comprehensive understanding of their structural viability.

## 1. Introduction

The motivation for this research is the practical significance of geopolymers, which are one of the most promising materials in the construction industry. Geopolymers are high-strength materials that compete in their properties with Portland cement, which is currently the main building material. During the production of 1 ton of Portland cement, 0.8 tons of CO_2_ are emitted into the atmosphere. Cement plants emit up to 1.5 billion tons of CO_2_ into the atmosphere annually. Possessing excellent environmental properties, geopolymers use recycled materials in their production. The specific research gap that the work aims to eliminate is as follows. In similar works of this type, the result of optimization of the composition of geopolymers is the establishment of the boundaries of change or ranges of variation in the obtained physical and mechanical properties of specific compositions, which is carried out empirically. Processing the results of experimental studies on the equality of the weight content of the criteria of physical and mechanical properties of various components of geopolymer matrices by determining the extremes also does not achieve the goal, since it requires a large amount of input information with a smaller discretization step. The extreme point can be missed in a limited volume of measured values. In multi-criteria optimization using the Pareto method, it is proposed that concessions be introduced to the main criterion, with an increase in the weight content of the rest. Although this procedure is statistically justified, it does not have an unambiguous physical interpretation when constructing an intelligent ranking system for optimality criteria when determining the composition of geopolymer matrices. Due to fundamental restrictions on the use of the model, a simultaneous change in two components has not been previously studied.

The research problem of geopolymer composites revolves around the imperative to comprehensively address their synthesis, structural performance, and environmental impact [1,2,3,4,5]. There is a pressing need to enhance our understanding of the intricate relationships between precursor characteristics, geopolymerization processes, and resulting material properties [6,7,8,9,10]. Additionally, investigating the long-term durability and structural behaviour of geopolymer composites under diverse environmental conditions is paramount. Bridging the gap in knowledge regarding the scalability of geopolymer production processes and their economic feasibility is essential for facilitating wider adoption in the construction industry [11]. The life cycle of the resulting composites is associated with the processes of changing their energy state and the formation and existence of edge and screw dislocations and internal friction mechanisms, the foundations of which are laid out in existing studies [12,13]. The continuity of the processes is due to averaging a large number of elementary interactions, which are accompanied by the release of free energy. In materials far from equilibrium, the evolution of dissipative structures occurs. Furthermore, assessing the environmental sustainability of geopolymer composites through life cycle analysis remains a critical research frontier. Waste generation leads to environmental pollution and depletion of natural resources, as it is a consequence of the unidirectional movement of natural formations used for producing products and subsequently converted into waste. Waste generation is the final stage in the life cycle of materials. Geopolymer materials are a reasonably reliable alternative to cement. At the same time, the possibility of introducing various additives into the structure of geopolymer compositions is of practical interest, especially in industrial waste, the disposal of which is an important scientific, technical, and environmental task. The possibility of reusing such a waste substance in the structure of geopolymer materials has not been previously studied in world practice. Such modified geopolymer materials are characterized by different disposal prospects determined by this type of substance’s physical, mechanical, and chemical properties. This multifaceted research problem encompasses the intricate interplay of material science, structural engineering, and environmental considerations, necessitating a holistic and integrated approach to propel geopolymer composites into mainstream construction practices.

Criterion methods are ways of describing alternative solutions based on quantitative expressions. Each decision leads to a certain result and therefore reflects the efficiency of the process and its value. In [14], the evaluation of the composition of the main power chains of an asphalt concrete mix based on discrete element methods is presented. In [15], a model for quantitative risk assessment of a structural hierarchical system used in the petrochemical industry is described. An algorithm based on optimality criteria for efficiently optimizing a layered composite design using simultaneous resizing and scaling is presented in [16]. The disadvantages of criterial methods are the need for the analyzed information to contain information on the direction of changes in properties, the execution of operations to normalize the data, ranking the information, and searching for matches corresponding to optimal parameters.

Markov chains are ways of describing probabilistic dynamics. Design-of-experiment methods have been widely studied for optimization of injection moulding. In [17], Bayesian adaptive design of experiment is proposed. Optimizing the electrical discharge machining process, which is essential for enhancing performance, is described in [18]. In [19], intelligent algorithms have been demonstrated to be effective in production scheduling, and thus, to enhance the efficiency of prefabricated component production scheduling. In [20,21], discrete Markov chains have been used to develop strategies in various industries, including in [22] in medical research, in [23] in the fight against coronavirus, in [24] in navigation systems, and in [25,26] in international relations. The disadvantages of Markov chains are expert assessment of probabilities and the lack of ranking of the alternatives used. The unification of semantically integrated technologies for processing information about the optimized structure and properties of geopolymers is possible based on the unification of criterial methods and Markov chains [21,27].

The information-entropic model for determining the necessary amount of information to determine the dynamics of changes in the main indicators of system development is presented in [28]. The use of fuzzy set theory and genetic algorithms for the creation of communication services is presented in [29]. Scientific research in this area is aimed mainly at improving technologies and expanding the range of their application, positioning geopolymer composites as an alternative material that is resistant to changes in the external environment, loads, pressure, and temperature fluctuations, and alternating effects. However, all of them are aimed at studying the behaviour of various material compositions of structural, strength, and environmental properties with the sequential introduction of various additives without taking into account their interactions, which is a complex mathematical problem. To some extent, it can be solved using a statistical approach, constructing a mathematical model in the form of a segment of the Taylor series, into which the unknown function is decomposed. However, the statistical approach requires a large number of experimental data for its implementation, which determines the accuracy of the method.

In the case of the statistical approach, the mathematical model of the object is represented in the form of a polynomial, i.e., a segment of a Taylor series into which the unknown function is decomposed. In the case of multilevel planning, the method of reducing the experiments by constructing orthogonal central composition plans is used. Here, the response function is a full nth degree function of the variables x_1_ and x_2_ [30].

For this purpose, it is necessary to go from a first-order polynomial considering only the first two coefficients to a second-order polynomial by checking the quality of the model description at the central point of the plan. The study of the methodology of constructing quadratic models of objects based on second-order plans and the theory of compositional planning is presented in [31]. In [32,33], a method of obtaining a trend equation in the form of a polynomial of the nth degree to describe the envelope of the acoustic emission signal is described, which was developed in the method of obtaining a polynomial regression equation using the orthogonal central composition plan.

One of the approaches for describing complex phenomena of resource optimizing based on similarity criteria is described in [34]. In [22,35,36], a multi-criteria method of optimizing complex systems using the example of a geopolymer matrix is described.

A multifactor cross-algorithm for resource provision for geopolymers operating in conditions of uncertainty is proposed in [37,38].

Multi-objective optimization, Markov chains, and design of experiment methods are relevant to the geopolymer industry but are poorly described.

The paper [39] summarizes recent developments in geopolymer research, focusing on raw material optimization, activator selection, durability assessment, and cost control. It is shown that there are still significant gaps in our knowledge. More confirmation of the long-term performance of geopolymers under different environmental conditions through field testing is needed. Moreover, variability in industrial by-products makes it difficult to standardize mixtures to obtain consistent mechanical properties. Future research should focus on refining activator formulations, improving mixing ratios, and incorporating reinforcement strategies to enhance mechanical strength and durability. Addressing these issues is critical for the wider adoption of geopolymer technology in sustainable construction. In [40], the optimal formulation developed for fast and low-temperature curing geopolymers consisting of –Si–O–Al– covalent bonds in a polymer network with additives including Ca(OH)_2_, fumed silica, and chopped carbon fibre was investigated. Multivariate formulation optimization was systematically conducted using experiments and metamodeling. Using the metamodel, an optimized geopolymer formulation was successfully developed in only 45 sets of experiments. In [41], an orthogonal experimental method is used to study the effects of the solid material ratio, amount of added water, alkali activator dosage, and alkali activator modulus on the mechanical properties and microstructure of geopolymers. In addition, single-factor experiments are conducted for two factors that have the greatest impact on the performance of geopolymers. Iron waste and metakaolin are used as the main raw materials, and liquid glass and NaOH are used as activators for the preparation of geopolymers. According to the above studies, the conditions for the preparation of geopolymers are optimized. In studies [42,43], a mixture of stabilized mine waste with different percentages of binders (e.g., metakaolin and pumice) and their effect on the mechanical, microstructural, and toxicological properties of the synthesized geopolymers were analyzed. The ratios of mine waste to binder varied from 100/0 to 0/100. The evaluations included compressive strength, chemical analysis using infrared spectroscopy, microstructural characterization, thermal behaviour in the temperature range from 40 °C to 1000 °C, and toxicological tests.

In [44], a systematic method that combines the statistical design of experiments, multiple response optimization method, and analytic hierarchy process for designing a geopolymer product is presented. The method is demonstrated using a case study involving a geopolymer made from a ternary mixture of red mud, rice husk ash, and diatomite. In addition to mechanical and thermal properties, production cost, energy consumption, and carbon footprint were considered when modelling the desirability of the product. The combined application of multi-criteria optimization methods, Markov chains, and a multi-factor mathematical, empirical model based on orthogonal central compositional second-order designs in the geopolymer industry presented in our study is an original and new direction of research, which has not been used in such a combination before.

The task of the research is to build a mathematical model for determining and predicting the optimal composition of the geopolymer matrix. In this case, a choice is made from a variety of physical and mechanical data characterizing the strength of geopolymer mixtures and obtained based on planned and conducted experiments. Experiment planning is a procedure for selecting several conditions and conducting experiments to obtain a mathematical model of the process. The aim is to minimize the number of experiments while varying all variables simultaneously.

The research objectives centre on elucidating the utilization of by-products from the secondary aluminum industry in geopolymer composite formulations. Hypotheses posit that tailored combinations of secondary aluminum by-products will enhance geopolymer reactivity and result in composites with superior mechanical strength and durability. Scientific and practical applications include optimizing the composition and properties of geopolymers, which are associated with the choice of their acceptable combination from a variety of physical and mechanical data characterizing the strength of geopolymer mixtures. In this case, not only the necessary composition of geopolymers, but also the choice of recommendations for determining the range of the main components is provided. The main difficulties in the modelling process of optimizing the technology for obtaining geopolymer materials with the specified properties are connected not so much with obtaining a huge volume of geopolymer compositions of further experimental studies as with processing of the obtained information, its generalization, and obtaining the necessary conclusions and recommendations.

The aim of the study is to develop a methodology for the simultaneous influence of various components of the structure of geopolymer compositions to obtain optimal physical, mechanical, thermal, and operational properties of materials.

The significance of the research lies in the practical focus on the use of secondary processing in the aluminum industry as a raw material for obtaining geopolymer compositions.

The potential advantage of this approach is to determine the formulation of a geopolymer mixture as a new structural material for the construction industry with the simultaneous interaction of the two following natural components: quartz sand and recycled waste from the aluminum industry. Existing methods for studying geopolymer materials are designed to study the effect of only one component with an unchanged content of others. The simultaneous influence of two components at once has not been studied before. This is the relevance of the work.

## 2. Materials and Methods

The inorganic two-component aluminosilicate binder, commercially known as Bausik LK and sourced from České lupkové závody, a.s., Nové Strašecí, Czech Republic, comprises metakaolin, with a grain size distribution of D50 = 3 µm and D90 = 10 µm. Activation is achieved through an aqueous alkaline activator. The manufacturer-prescribed mixing ratio dictates the preparation of the binder mixture based on the inorganic polymer, typically employing five parts by weight of metakaolin and four parts of the activator. In the formulation, silica sand, denoted as ST 01/06 from Sklopísek Střeleč, a.s., Újezd pod Troskami, Czech Republic, is utilized as an aggregate, characterized by a grain size distribution of D50 = 0.44 and D90 = 0.63 mm. Furthermore, the composite is reinforced with chopped carbon fibres possessing an elastic modulus of up to 230 GPa and a tensile strength of 3500 MPa. The mineralogical composition of C.FG encompasses aluminum calcite, rutile, graphite, and ankerite. The C.FG creates a porous geopolymer structure. The particle size of these C.FG materials ranges from 0.40 to 355.66 µm, with an elemental analysis indicating aluminum concentrations of 73.296 ppm. Detailed information about the characteristics and properties of the C.FG material contributes to a nuanced understanding of these fillers and their potential impact on the overall composition and performance of geopolymers within the context of the secondary aluminum industry.

### 2.1. Preparation of Samples

The synthesis process of the geopolymer composites involved the meticulous amalgamation of metakaolin and the alkaline activator for approximately 5 min, ensuring the attainment of a homogenous mortar. For additives from the aluminum industry waste in the structure of the geopolymer composition, the raw material was first crushed, ground into dust, and then added to the geopolymer mixture formulation. Subsequently, chopped carbon fibres were introduced into the mixture, and the amalgamation continued for an additional 2 min. Following this, silica sand was incorporated into the composite, and the blending continued for 3 min. Finally, industrial by-product material was integrated into the composition, with a mixing duration of 2 min, facilitating the preparation of diverse geopolymer composites.

**Figure 1:**
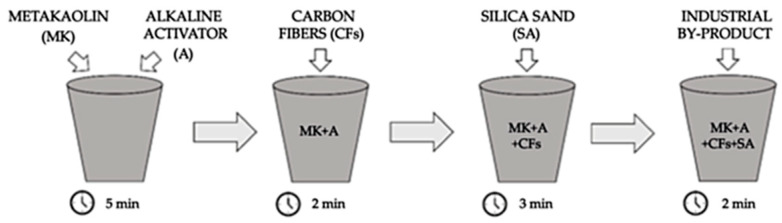
The preparation process of the test samples [10].

After mixing, the geopolymer mortar was carefully poured into moulds with the required test specimen size. These specimens were subsequently enveloped with polypropylene film and left to cure at room temperature for an incubation period of approximately 24 h. Following this curing duration, the samples were de-moulded, rewrapped with polypropylene film, and allowed to mature at room temperature for an additional 28 days before undergoing comprehensive analytical assessments. The experimental procedure detailed in Figure 1 illustrates the mixing process of raw materials used to prepare the test samples.

### 2.2. Measurements and Characterization

**Figure 2:**
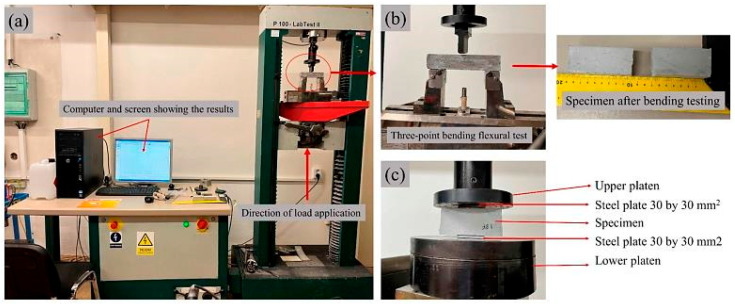
Stress testing setup: (**a**) Instron model 4202 testing machine, (**b**) bending testing setup, (**c**) compression testing setup.

**Figure 3:**
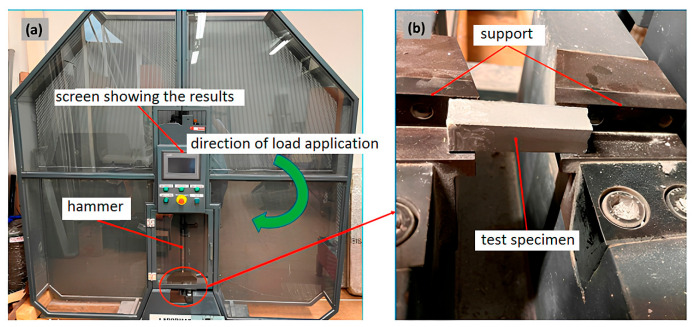
Charpy impact test setup: (**a**) universal testing machine, (**b**) specimen testing setup.

The three-point bending tests were executed utilizing an INSTRON Testing Machine (Model 4202) INSTRON, Norwood, MA, USA, adhering to standard [45]. Six specimens with dimensions of 30 × 30 × 150 mm^3^ were subjected to the tests at room temperature, employing a crosshead speed of 6.0 mm/min and a span length of 100 mm. To determine the flexural strength, subsequent compressive tests were conducted using the same INSTRON Testing Machine, following standard [46] (Figure 2 and Figure 3). The broken parts from the specimens utilized in the bending test were repurposed to create twelve samples measuring 30 × 30 × 30 mm^3^ each. These compressive tests were conducted at room temperature, employing a crosshead speed of 6.0 mm/min. 

For impact testing, a PIT-C Series Pendulum Impact Testing Machine, Tinius Olsen, Horsham, PA, USA adhering to standard [47] was employed, featuring a pendulum capacity of 150 J, and energy loss compensation of 0.23 J, and an estimated absorbed energy of 150 J. Six specimens with dimensions of 19 × 20 × 60 mm^3^ underwent testing at room temperature.

Thermal analyses were tested after 28 days of curing with dimensions of 150 × 150 × 30 mm^3^; each specimen underwent six measurements using the Isomet 2114 device, Applied Precision Ltd., Bratislava, Slovakia following standard [48]. This microprocessor-controlled commercial instrument, equipped with interchangeable probes, facilitated comprehensive thermal analysis. Geopolymer density was determined by dividing the sample mass by volume, adhering to standard [49].

### 2.3. Method of Multicriteria Optimization

The optimal formulation of geopolymer composition implies maximization of compressive strength, bending strength, impact toughness, and minimisation of density and thermal conductivity values.

When conducting research in several cases, when the investigated objects or processes are excessively complex and the understanding of the mechanism of processes is insufficient, it is impossible to construct analytical (deterministic) dependencies based on the phenomena of transfer or balance of different quantities. Such problems can be solved by constructing a multifactor mathematical empirical model based on orthogonal central composition plans for the second order.

**Figure 4:**
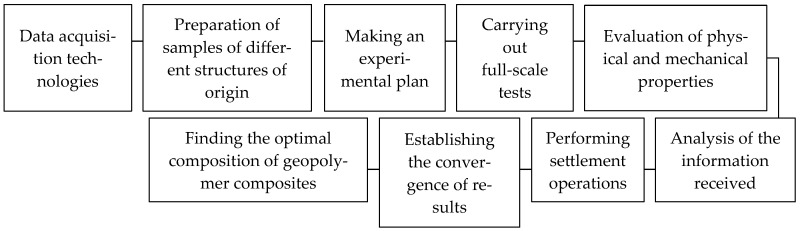
Research flow chart.

The flow chart of the research is presented in Figure 4.

In this study, Markov chains, as applied to the research problem, represent a tool of the theory of random processes consisting of a sequence of states of structure formation of geopolymer composites. This is interpreted as the sum of a series of probabilities of a certain event *K*, depending on the occurrence of the state *I*, where the events are *K* = *m* + 1, *I* = *m*, i.e., event K differs from *I* by 1

(1) $$\sum_{k=1}^{r}p_{ik}=\sum_{k=1}^{r}P(X_{m+1}=k|X_{m}=i),$$

The probability distribution in the Markov chain depends only on the transitions from the current state to the next. Therefore, the Markov chain is represented as a sequence of states of formation of the structure of geopolymer mixtures when adding aluminum raw material processing products. Mathematically, this looks like this

(2) $$X={\left(X_{n}\right)}_{n\in N}=(X_{0},X_{1},X_{2},\ldots ),$$

where at each moment of time, the process of structure formation takes such values in a discrete set that *X_n_* ∈ *E* and ∀*_n_* ∈ *N*

In the work, Markov chains combine a priori assumptions and experimental data to obtain a posteriori distribution of the parameter of interest.

Multiplying the probability distribution at a certain stage of determining the composition of geopolymer compositions by the matrices of transition probabilities, we obtain the probability distribution at the next stage of formation of the structure of the geopolymer composition when adding processed aluminum raw materials.

When establishing criterion assessments, it is necessary to choose guidelines that maximally cover the conditions for implementing the set goal. For this, it is necessary that all options for changing the physical, mechanical, and thermal properties of the studied structure of the geopolymer composition be compatible with the dimensions and directions of the structure. To compare possible alternatives for the components of the formulation of geopolymer mixtures, each of them is assigned a point, and the final assessment is made on a rank scale. Existing methods for assessing the composition of a geopolymer mixture do not take into account the diversity of empirical observations. This reduces the effectiveness of optimization methods for multiple criteria, so their modernization is necessary, which consists of the transition from vector optimization to a scalar. Decision-making for the composition of a geopolymer mixture begins with the construction of a matrix *R*.

(3) $$R=\left(\begin{array}{ccccc} & \Pi_{1}\; & \Pi_{2} & \ldots & \Pi_{j}\\ q_{1} & \delta y_{11} & \delta y_{12} & \ldots & \delta y_{1j}\\ q_{2} & \delta y_{21} & \delta y_{22} & \ldots & \delta y_{2j}\\ \ldots & \ldots & \ldots & \ldots & \ldots \\ q_{i} & \delta y_{i1} & \delta y_{i2} & \ldots & \delta y_{ij} \end{array}\right),$$

where *q*_1_…*q_i_*—number of samples with a certain content of components in the geopolymer mixture, Π_1_…Π_j_—physical–mechanical and strength parameters of geopolymer, *i*—line number, *j*—column number, *δy_ij_*—the relative deviation of the trait from the optimal value.

Target parameters of optimization select maximum minimum or average values; however, in this case, coincidences with experimental values are possible. To expand the boundaries of features, knowledge of the magnitude of the weight coefficients is required, which we determined based on the results of calculating Markov chains. Therefore, it is important to study the influence of the properties of target functions. The main criteria are the Savage (Wald) criterion

(4) $$W=\underset{1\leq i\leq m}{\max}\underset{1\leq j\leq n}{\min}\delta y_{ij},$$

Laplace criterion

(5) $$L=\underset{1\leq i\leq m}{\max}\underset{1\leq j\leq n}{\max}\delta y_{ij},$$

and the Hurwitz criterion

(6) $$H_{z}=\underset{1\leq i\leq m}{\max}\{\rho \underset{1\leq j\leq n}{\min}\delta y_{ij}+(1-\rho )\underset{1\leq o\leq m}{\max}\delta y_{ij}\}$$

The Savage criterion considers unfavourable situations when a decision is made without the possibility of correcting the situation. The Laplace criterion does not take into account possible negative ways of developing situations. The Hurwitz criterion is the basis for decision-making when establishing ranges between. The main stages of construction of the system of optimization of the composition of geopolymer mixture are experimental research, mathematical calculations, and interpretation of results.

Planning an experiment to obtain the regression equation in the form of a quadratic polynomial is called second-order planning. The second-order polynomial contains summands that account for main linear effects, pairwise interaction effects, and quadratic effects. To avoid difficulties related to the difference in the dimensionality of factors, it is necessary to convert the natural values of factors into dimensionless quantities (coding of factors) using the following formula:

(7) $$x_{j}=\frac{X_{j}-X_{j0}}{h_{j}},$$

where *x_j_*—coded value of the *j*-th factor; *X_j_*—natural (dimensional) value of the *j*-th factor; *X_j_*_0_—natural (dimensional) value of the main (average) level of the *j*-th factor; *h_j_*—step of variation in the *j*-th factor.

After coding the factors, the polynomial will have the following general form:

(8) $$\tilde{y}=\sum_{j=0}^{m}b_{j}x_{j}+\sum_{j=1}^{m}\sum_{k=j+1}^{m}b_{jk}x_{j}x_{k}+\sum_{j=1}^{m}b_{jj}x_{j}^{2},$$

where *m*—number of factors.

The first summand of the right-hand side of Equation (8) accounts for main linear effects, the second for interaction effects, and the third for quadratic effects (all in coded form).

The number of coefficients *b_jk_* of the quadratic regression equation to be determined is

(9) $$n_{k}=\frac{m^{2}+3m+2}{2}$$

To solve the above problems, an experiment conducted according to a second-order orthogonal central composition plan (OCCP) is needed, in which the number of experiments is determined by the following formula:

(10) $$n=2^{m}+2m+1$$

The planning matrix for the two-factor experiment (*m* = 2) is given in Table 1.

A dummy factor has been introduced in Table 1 *x*_0_ = 1, so that a regression equation in the form of Equation (8) can be used. The experimentally measured and varied factors are as follows: *x*_1_, *x*_2_ (two-factor experiment) and values of *x*_3_, *x*_4_, and *x*_5_ are calculated from experimental results and take into account interaction effects and quadratic effects, respectively.

The core of the orthogonal central composition plan is a full-factor experiment, supplemented by a *m* pairs of symmetrical points located on the coordinate axes at some distance from the plan centre (star points), as well as a point in the plan centre (zero point). The number α is called the star arm.

To ensure the symmetry and orthogonality of the plan, the quadratic effects are taken into account by introducing the correction *φ* and fictitious factors *x_j_*^2^ − *φ,* where *φ* is a positive constant number called the quadratic correction.

The correction value *φ* and a starry shoulder *α* are uniquely determined based on the conditions of symmetry and orthogonality of the plane by solving the system of equations.

(11) $$\left\{\begin{array}{c} 2^{m}\left(1-\phi \right)+2\left(\alpha^{2}-\phi \right)-\left(2m-1\right)\phi =0\\ 2^{m}{\left(1-\phi \right)}^{2}-4\phi \left(\alpha^{2}-\phi \right)+\left(2m-3\right)\phi^{2}=0 \end{array}\right.$$

The solution of the above system of equations for the two-factor experiment (*m* = 2) gives the following values: *α* = 1, *φ* = 2/3.

Given the orthogonality of the planning matrix, the coefficients of the regression equation are determined by the following formula:

(12) $$b_{jk}=\frac{\sum_{i=1}^{n}{\left(y_{i}x_{jk}\right)}_{i}}{\sum_{i=1}^{n}{\left(x_{jk}\right)}_{i}^{2}},$$

where *y_i_* is the response in the *i*-th experiment (the result of the experiment),

(13) $$x_{jk}=\left\{\begin{array}{ll} x_{j}x_{k} & j=0,\;j\neq k\\ x_{j}^{2}-\phi & j\neq 0,\;j=k \end{array}\right.$$

Thus, regression Equation (8), taking into account quadratic effects for the two-factor experiment, is written as follows:

(14) $$\tilde{y}=b_{0}x_{0}+b_{1}x_{1}+b_{2}x_{2}+b_{12}x_{1}x_{2}+b_{11}\left(x_{1}^{2}-\phi \right)+b_{22}\left(x_{2}^{2}-\phi \right)$$

The main components used for the synthesis of geopolymer foams are metakaolin, alkaline activator, silica sand, carbon fibres, and C.FG. As two varying factors, we assume that *X*_1_ is.FG, *X*_2_ is silica sand, and the other components of the geopolymer matrix are kept constant. The variation limits *X_j_*_min_ and *X_j_*_max_, mean values *X_j_*_0_, and variation step *h_j_* for each factor are presented in Table 2.

Based on the orthogonal central composition plan for the two-factor experiment (Table 1) and the limits of variation in factors (Table 2), the plan of experiment in natural values of factors was made. The results of this planning are presented in Table 3.

True value of the coefficients *b_jk_* lies in the interval [*b_jk_* − Δ*b_jk_*, *b_jk_* + Δ*b_jk_*]. This statement is true with some reliable probability *P*.

The value ∆*b_jk_* is found using the formula

(15) $$\Delta b_{jk}=t\sqrt{\sigma_{bik}}$$

where *t*—Student’s coefficient, *σ_bik_*—dispersion determined by the formula

(16) $$\sigma_{bjk}=\frac{1}{{\left(\sum_{i=1}^{n}{\left(x_{jk}\right)}_{i}^{2}\right)}^{2}}\sum {\left(x_{jk}\right)}_{i}^{2}\sqrt{\frac{{\left(y_{i}-{\tilde{y}}_{i}\right)}^{2}}{n\left(n-1\right)}}$$

where *y_i_*—experimental value of the parameter, ${\tilde{y}}_{i}$—theoretical value of the parameter.

## 3. Results and Discussion

The first stage of the study was the production of nine samples of geopolymers, and the content of components was determined according to the developed plan (Table 3). After each sample, measurements of the following physical and mechanical properties were carried out: compressive strength, bending strength, impact strength, density, and thermal conductivity. The results of these measurements are presented in Table 4.

Table 4 presents data collected from the results of laboratory measurements of physical, mechanical, and thermal properties performed on samples, taking into account the possible ranges of concentrations of the components studied.

Since the objective of this study is to construct a second-order mathematical model to determine and predict the optimal geopolymer matrix composition, the obtained set of values of five physical and mechanical properties was subjected to a multi-criteria optimizing procedure.

The procedure for determining the optimal geopolymer composition based on the analysis of physical and mechanical parameters begins with the construction of the efficiency matrix (3).

The relative deviation *δy_ij_ j*-th of the trait from the optimal value is determined as follows.

(17) $$\delta y_{ij}=\left\{\begin{array}{c} \frac{\left|y_{ij}-c_{j}\right|}{y_{j,\max}-c_{j}};\;y_{ij}>c_{j}\\ \frac{\left|y_{ij}-c_{j}\right|}{c_{j}-y_{j,\min}};\;y_{ij}<c_{j} \end{array}\right.,$$

For parameters *c_j_*, it is necessary to choose the best values of the analyzed parameters from the point of view of the problem to be solved—these are the maximum values of compressive strength, bending strength, impact toughness, and the minimum values of density and thermal conductivity from the experimental sample. With this approach, Formula (15) will convert the dimensional values into relative values within the scale (0, 1). However, with this choice of parameters, *c_j_* will necessarily be observed coinciding with the value of the *c_j_* corresponding elements of matrix (3), which will lead to *δy_ij_* = 0. When using additive convolution (16), this leads to the dropping of the corresponding attribute from the overall evaluation of the object. An obvious way to avoid such situations is to extend the upper (for maximum) or lower (for minimum) limit of each attribute *c_j_* in the same percentage ratio. Below are the maximum (minimum) values of each of the analyzed parameters *c_j_*, which increased (decreased) by 1%.

To select the optimal combination of geopolymer matrix components, not one but several criteria must be taken into account. Therefore, a convolution of such multidirectional criteria is necessary.

A generalized multi-criteria utility function—additive convolution—was used in the theoretical analysis:

(18) $$y_{a}=\delta y_{i}=\sum_{j=1}^{n}\omega_{j}\delta y_{ij},$$

where *ω_j_* is the weight coefficient of the *j*-th attribute.

And the geopolymer matrix composition with the minimum value of function (17) is considered to be the best. As a response function, it is better to take the value of

(19) $$y=1-y_{a}=1-\sum_{j=1}^{n}\omega_{j}\delta y_{ij},$$

At such a choice of response function, its maximum value will correspond to the best composition of the geopolymer matrix. Thus, the degree of remoteness of the current value of the response function for a particular geopolymer matrix composition from its maximum possible value will characterize the degree of optimality of this composition.

The priority for the arrangement of conditional probabilities (weight coefficients *ωj*) and physical and mechanical properties was determined based on experimental estimates of the role of this parameter in the mechanism of geopolymer structure formation, taking into account that the sum of conditional probabilities in the row of the matrix is equal to one. The columns of the conditional probability matrix characterize the number of iterations and transitions of the system during its evolutionary transformations and should not be summed.

At the same time, it is envisaged to set the weighting coefficients in the form of a probability distribution over all admissible values of Markov chains. This approach allows taking into account the a priori distribution of the overall output parameters and calculating the posterior distribution using the generated assumptions.

The construction of Table 5 starts with filling its first row based on expert judgments and a priori knowledge about the subject area. Formalization of the presented knowledge in the form of Table 2 parameters is implemented through logical inference and evolution of iteration steps.

Each state of the parameters characterizing the information situation of determining the physical and mechanical properties of geopolymers at a given formulation of their production is assigned a certain probability, recorded as a row of the matrix of states. The matrix of intensities or transitions of the system describes the wandering of the system through its states. In the process of analyzing the state matrix, all possible states of the parameters are renumbered with their probabilities, i.e., we are dealing with a set of probability values between iterations.

The initial state vector in Table 5 can be written in the following form:

(20) P(0)=[0.300, 0.260, 0.180, 0.160, 0.100]

The matrix of transition probabilities according to Table 5 has the following form:

(21) $$T=\left[\begin{array}{ccccc} 0.300 & 0.260 & 0.180 & 0.160 & 0.100\\ 0.330 & 0.240 & 0.160 & 0.140 & 0.130\\ 0.340 & 0.180 & 0.140 & 0.140 & 0.200\\ 0.310 & 0.220 & 0.160 & 0.130 & 0.180\\ 0.290 & 0.240 & 0.150 & 0.160 & 0.160 \end{array}\right]$$

Multiplying the initial state vector *P*(0) by the transition probability matrix *T*, we obtain the probability distribution at the first decision stage *P*(1). According to the method of calculating Markov chains, this probability will be equal to

(22) P(1)=P(0)×T=[0.316, 0.232, 0.161, 0.146, 0.145]

Multiplying the state vector *P*(1) by the transition probability matrix *T*, we obtain the probability distribution of the next decision step *P*(2).

(23) P(2)=P(1)×T=[0.313, 0.234, 0.162, 0.148, 0.143],

Repeating the described iterations, we obtain the probability distribution of the next decision stages:

(24) P(3)=P(2)×T=[0.313, 0.233, 0.162, 0.148, 0.144],

(25) P(4)=P(3)×T=[0.313, 0.233, 0.162, 0.148, 0.144]

It follows from the theory of Markov chains that the shorter the cycle length, the more accurate analyses can be performed. As seen, starting from the third step, the probabilities have arrived at a stationary state, in which the probability values correspond to the weight coefficients of the parameters for optimizing the strength properties: compressive strength 0.313, bending strength 0.233, impact strength 0.162, density 0.148, thermal conductivity 0.144.

Relative deviations calculated according to Formula (17) physical and mechanical characteristics, *δy_ij_*, of geopolymers from the optimal value, as well as the value of additive convolution (18) and response function, are presented in Table 6. The calculations were carried out under the assumption that all criteria have different importance, defined above with the help of Markov chains.

Now, we can finally form the orthogonal central composition matrix to calculate the coefficients of the regression equation and build a second-order mathematical model to determine and predict the optimal composition of geopolymer material. The results are presented in Table 7.

The values of the regression equation coefficients calculated by Formula (12) are given in Table 8.

Thus, the regression equation can be written in its final form:

(26) $$\tilde{y}\left(x_{1},x_{2}\right)=0.452+0.060x_{1}+0.031x_{2}+0.013x_{1}x_{2}-0.189\left(x_{1}^{2}-0.667\right)-0.079\left(x_{2}^{2}-0.667\right)$$

Table 7 also shows the results of the comparison of measured y and predicted by the model (calculated) $\tilde{y}$ responses. The accuracy of the model was assessed based on the calculation of the non-convexity Δ and relative error *ε*:

(27) $$\Delta =\left|y-\tilde{y}\right|,$$

(28) $$\varepsilon =\frac{\Delta }{y}$$

The analysis of the model errors shows that the maximum value of the relative error is 23.4%, and the minimum value is 3.1%. Therefore, the correspondence of the theoretical model to the experimental data can be considered acceptable.

The absolute errors in determining the regression coefficients were calculated according to Equations (15) and (16) for the Student’s coefficient *t* = 2.3, which corresponds to a confidence probability of *P* = 0.95. The results are presented in Table 8.

The maximum function (20) is observed at the following values of coded factors:

$$x_{1}=0.166;\;x_{2}=0.211$$

which corresponds to the best composition of the geopolymer matrix and in dimensional units

$$X_{1}=C.\mathrm{FG}=62.493\;g;\;X_{2}=\mathrm{Silica}\;\mathrm{sand}=1052.826\;g$$

**Figure 5:**
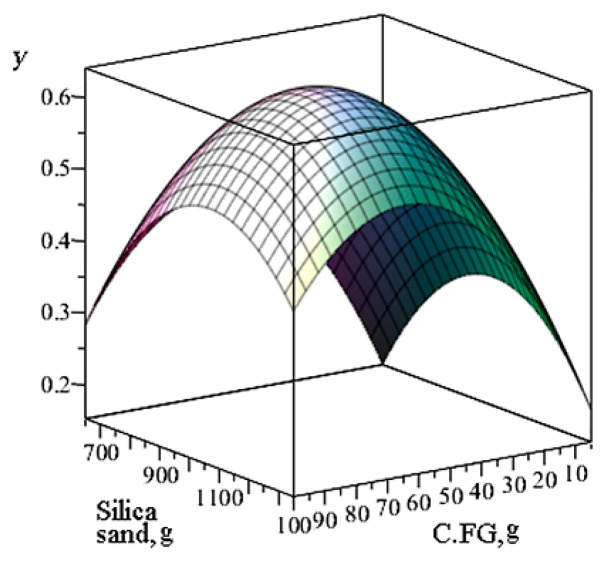
Response surface of the model for determining and predicting the optimal composition of geopolymer material.

Thus, the obtained mathematical model allows for the accurate determination of the optimal content of two components of the geopolymer matrix—C.FG and silica sand. Figure 5 shows the response surface of the model.

**Figure 6:**
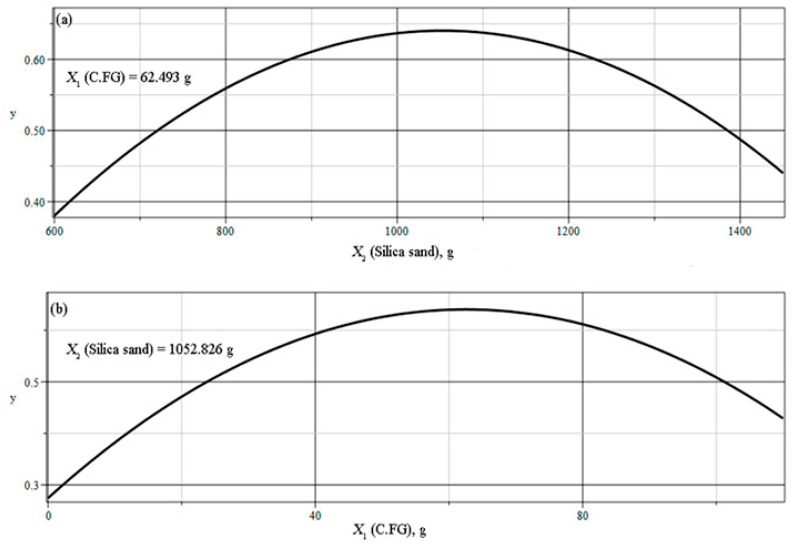
Cross-sections of the model response surface at: (**a**) optimal fixed value of factor X_1_ (C.FG), (**b**) optimal fixed value of factor X_2_ (silica sand).

Figure 6 shows the cross sections of the response surface of the model at optimal fixed values for one of the factors.

Mathematical processing of statistical information on the properties of the studied composite materials and technologies for their production using multi-criteria optimization tools and plans for constructing and obtaining experimental information allows for reducing the labour intensity of computational operations and ensuring the completeness of their purposefulness. At the same time, it provides the possibility of obtaining hidden patterns and trends that simple methods of mathematical statistics cannot detect.

The analysis of the influence of various by-products of the aluminum industry on the geopolymerization process showed the suitability of the proposed methods of mathematical processing of results for assessing the mechanical, thermal, and environmental properties of the resulting composites to create a comprehensive idea of their structural viability. From the point of view of possible utilization of the resulting geopolymer substance, it can be considered from two positions as thermoplastic and thermosetting. The thermosetting component does not raise doubts due to the low reactivity of the components used: metakaolin, alkaline activator, carbon fibres, and silica sand.

The issues of studying the properties of geopolymer compositions obtained with the addition of quartz sand and recycled aluminum waste are discussed in detail in our previous works devoted to the study of technologies and the properties of using aluminum industry waste processing products as raw materials for obtaining geopolymer materials [9,10].

It has been established that the reuse of waste when heating the geopolymer composition causes a weakening of physical bonds while chemical covalent bonds are preserved; therefore, the chemical structure of the materials remains unchanged. It has been shown that the utilization of the resulting geopolymer composition is a technologically simple process that allows for increasing the degree of reuse of substances and materials and thereby ensuring the rational use of natural resources. At the same time, this makes it possible to organize the production of some products used as raw materials for the utilized geopolymer composition in, for example, shipbuilding, ship repair, and coastal water transport facilities.

The novelty and uniqueness of the proposed work lies in the use of an object-oriented approach applied to reduce the number of experiments in two-level planning. Existing methods for determining the required composition of composite mixtures are based on a sequential increase in the content of the component under study with synchronous observation of changes in the obtained properties with an unchanged content of other components. Such methods require a large number of experimental studies. In the case of a simultaneous change in two components of the mixture, such a problem becomes unsolvable even with a huge number of experiments, since it is very difficult, and sometimes impossible, to interpret the results obtained. The content of the information lies in the analysis of interacting objects, which are the components of the geopolymer mixture. They form an information space, the morphology of which organizes the planning structure for determining the concentrations of the components of the geopolymer mixture. The uniqueness of the presentation of the results lies in highlighting the nature and features of the interaction of the main competing elements of the geopolymer recipe and translating the model of their harmonic relationships. The purpose of the proposed method is to identify system objects in the form of concentrations of the elements under study and distribute the ratios between them in the required accordance to impart the corresponding properties to the geopolymer mixture.

The computational basis for the calculations was the digitalization of the technology for studying and analyzing the methodology for the complex interaction of two components of a geopolymer mixture of quartz sand and by-products of the aluminum industry.

The practical originality of the work is the use of by-products from the aluminum industry as components of secondary processing, which is also an essential element of the novelty of the presented work, due to this achieved optimization of mechanical, thermal, and physical properties.

The optimal composition of the structural elements of geopolymer mixtures for the given technology of their production has been determined. The experiments and calculations have shown that the use of the research results in conjunction with the optimization criterion is the basis for increasing the reliability of the evaluation of process parameters and optimization of the properties of mixtures, determining the composition and structure of materials when changing the technologies of their production. Modelling the parameters of the objective function showed the advantage of digitalization of technology during the analysis of the properties of geopolymer mixtures and their mathematical processing.

## 4. Conclusions

The presented model of geopolymer foam production technology for two-component variability of geopolymer matrix composition allows us to determine the formulation point where the proportions of each component in the geopolymer matrix are optimally combined. The presented mathematical model can serve as a reliable tool for controlling the structure formation of two-component complex systems.

The advantage of the developed optimization model is the possibility of visualizing the dynamics of the relationship between the responses to changes in the studied structural components.

The study’s findings may inform industry practices by offering a novel avenue for the utilization of secondary aluminum by-products, promoting resource efficiency and waste valorisation.

The study’s significance extends to its potential to drive innovation in the construction sector, promoting the adoption of greener technologies and practices.

## Figures and Tables

**Table 1:** Matrix of the orthogonal central composition plan when *m* = 2.

*i*	*x*_0_	*x*_1_	*x*_2_	*x*_3_ = *x*_1_*x*_2_	*x*_4_ = *x*_1_^2^ − φ	*x*_5_ = *x*_2_^2^ − φ
1	+1	+1	+1	+1	1 − *φ*	1 − *φ*
2	+1	−1	+1	−1	1 − *φ*	1 − *φ*
3	+1	+1	−1	−1	1 − *φ*	1 − *φ*
4	+1	−1	−1	+1	1 − *φ*	1 − *φ*
5	+1	+*α*	0	0	*α*^2^ − *φ*	− *φ*
6	+1	−*α*	0	0	*α*^2^ − *φ*	− *φ*
7	+1	0	+*α*	0	−*φ*	*α*^2^ − *φ*
8	+1	0	−*α*	0	−*φ*	*α*^2^ − *φ*
9	+1	0	0	0	−*φ*	−*φ*

**Table 2:** Natural values of the levels of the two factors and their variation steps.

Factors		*X*_1_ = C.FG	*X*_2_ = Silica Sand
Units of measurement of factors		gram	gram
Steps for varying the factors *h_j_*		45	250
Factor levels	*X_j_*_min_	10	750
	*X_j_*_0_	55	1000
	*X_j_*_max_	100	1250

**Table 3:** Natural values of factors in the two-factor experiment based on the orthogonal central composition plan.

By Wt. (Gram)					
Metakaolin	Alkaline Activator	*X*_2_ = Silica Sand	Carbon Fibres	*X*_1_ = C.FG	ID Samples
1000	900	1250	10	100	1
		1250		10	2
		750		100	3
		750		10	4
		1000		100	5
		1000		10	6
		1250		55	7
		750		55	8
		1000		55	9

**Table 4:** Mechanical properties of all samples.

ID Samples	Density, *ρ* [g/cm^3^]	Bending Strength, *σ_f_* [MPa]	Compressive Strength, *σ_c_* [MPa]	Impact Strength, *σ_i_* [MPa]	Thermal Conductivity, *λ* [W/mK]
1	1.261	2.250	6.640	0.330	0.501
2	1.095	2.060	3.900	0.330	0.421
3	1.261	2.210	6.570	0.260	0.516
4	1.091	2.070	3.910	0.270	0.432
5	1.110	2.230	4.050	0.350	0.444
6	1.250	2.090	6.670	0.280	0.522
7	1.116	2.110	6.170	0.310	0.51
8	1.118	2.190	6.370	0.330	0.51
9	1.060	2.580	4.940	0.310	0.415

**Table 5:** Conditional probabilities of optimality criteria.

Subsequent State Current State	Compressive Strength	Bending Strength	Impact Strength	Density	Thermal Conductivity
Compressive strength	0.300	0.260	0.180	0.160	0.100
Bending strength	0.330	0.240	0.160	0.140	0.130
Impact strength	0.340	0.180	0.140	0.140	0.200
Density	0.310	0.220	0.160	0.130	0.180
Thermal conductivity	0.290	0.240	0.150	0.160	0.160

**Table 6:** Matrix of relative deviations physical and mechanical properties, *δy_ij_*, from the optimal values, as well as the value of additive convolution and response.

ID Samples	Density *ρ*	Bending Strength σ_f_	Compressive Strength *σ_c_*	Impact Strength *σ_i_*	Thermal Conductivity *λ*	*y_a_*	*y* = 1 − *y_a_*
1	1.000	0.651	0.034	0.251	0.816	0.468	0.532
2	0.215	1.000	1.000	0.251	0.089	0.631	0.369
3	1.000	0.725	0.058	1.000	0.944	0.633	0.367
4	0.196	0.981	0.996	0.893	0.187	0.741	0.259
5	0.286	0.688	0.947	0.037	0.298	0.548	0.452
6	0.948	0.945	0.023	0.786	1.000	0.639	0.361
7	0.314	0.908	0.199	0.465	0.908	0.526	0.474
8	0.324	0.761	0.129	0.251	0.926	0.440	0.560
9	0.050	0.047	0.633	0.465	0.037	0.297	0.703

**Table 7:** Matrix of orthogonal central composition plan for building a mathematical model for determining and predicting the optimal composition of geopolymer material and the results of its application.

ID Samples	Matrix Elements						Comparison of Results			
	Factors						Responses (Experiment, Theory)		Errors	
*i*	*x*_0_	*x*_1_	*x*_2_	*x*_3_ = *x*_1_*x*_2_	*x*_4_ = *x*_1_^2^ − φ	*x*_5_ = *x*_2_^2^ − φ	*y*	$\tilde{y}$	Δ	*ε*
1	+1	+1	+1	+1	0.333	0.333	0.532	0.468	0.064	0.118
2	+1	−1	+1	−1	0.333	0.333	0.369	0.320	0.049	0.130
3	+1	+1	−1	−1	0.333	0.333	0.367	0.378	0.011	0.031
4	+1	−1	−1	+1	0.333	0.333	0.259	0.285	0.026	0.102
5	+1	+1	0	0	0.333	−0.667	0.452	0.502	0.050	0.112
6	+1	−1	0	0	0.333	−0.667	0.361	0.382	0.021	0.058
7	+1	0	+1	0	−0.667	0.333	0.474	0.583	0.011	0.234
8	+1	0	−1	0	−0.667	0.333	0.560	0.521	0.039	0.068
9	+1	0	0	0	−0.667	−0.667	0.703	0.632	0.071	0.100

**Table 8:** Values of the regression equation coefficients of the mathematical model for determining and predicting the optimal composition of geopolymer materials.

*b*_00_	*b*_01_	*b*_02_	*b*_11_	*b*_12_	*b*_22_
0.452	0.060	0.031	−0.189	0.013	−0.079
Δ*b*_00_	Δ*b*_01_	Δ*b*_02_	Δ*b*_11_	Δ*b*_12_	Δ*b*_22_
0.009	0.001	0.002	0.007	0.003	0.002

## Data Availability

The original contributions presented in this study are included in the article. Further inquiries can be directed to the corresponding authors.
